# Alcohol consumption and risky sexual behaviour in the fishing communities: evidence from two fish landing sites on Lake Victoria in Uganda

**DOI:** 10.1186/1471-2458-12-1069

**Published:** 2012-12-11

**Authors:** Nazarius M Tumwesigye, Lynn Atuyambe, Rhoda K Wanyenze, Simon PS Kibira, Qing Li, Fred Wabwire-Mangen, Glenn Wagner

**Affiliations:** 1 School of Public Health, Makerere University College of Health Sciences, Kampala City, Uganda; 2 Center for Behavioural Epidemiology and Community Health, San Diego State University, San Diego, USA; 3 Rand Corporation, Santa Monica, USA

**Keywords:** Risky sexual behaviour, HIV, AIDS, Alcohol consumption, Hazardous drinking, Harmful drinking, Harmful use of alcohol

## Abstract

**Background:**

The fishing communities are among population groups that are most at risk of HIV infection, with some studies putting the HIV prevalence at 5 to 10 times higher than in the general population. Alcohol consumption has been identified as one of the major drivers of the sexual risk behaviour in the fishing communities. This paper investigates the relationship between alcohol consumption patterns and risky behaviour in two fishing communities on Lake Victoria.

**Methods:**

Face-to-face interviews were conducted among 303 men and 172 women at the fish landing sites; categorised into fishermen, traders of fish or fish products and other merchandise, and service providers such as casual labourers and waitresses in bars and hotels, including 12 female sexual workers. Stratified random sampling methodology was used to select study units. Multivariable analysis was conducted to assess independent relationship between alcohol consumption and sexual risky behaviour. Measures of alcohol consumption included the alcohol use disorder test score (AUDIT), having gotten drunk in previous 30 days, drinking at least 2 times a week while measures for risky behaviour included engaging in transactional sex, inconsistent condom use, having sex with non-regular partner and having multiple sexual partners.

**Results:**

The level of harmful use of alcohol in the two fishing communities was quite high as 62% of the male and 52% of the female drinkers had got drunk in previous 30 days. The level of risky sexual behaviour was equally high as 63% of the men and 59% of the women had unprotected sex at last sexual event. Of the 3 occupations fishermen had the highest levels of harmful use of alcohol and risky sexual behaviour followed by service providers judging from values of most indicators. The kind of alcohol consumption variables correlated with risky sexual behaviour variables, varied by occupation. Frequent alcohol consumption, higher AUDIT score, having got drunk, longer drinking hours and drinking any day of the week were strongly correlated with engaging in transactional sex among fishermen but fewer of the factors exhibited the same correlation among traders and service providers. Fishermen who drank 2 or more times a week were 7.9 times more likely to have had transactional sex (95% CI: 2.05-30.24) compared to those who never drank alcohol. A similar pattern was observed for traders and service providers at the landing sites. Inconsistent condom use or none use of condoms was not significantly correlated with any of the alcohol consumption indicator variables in multivariate analysis except for day of drinking among men.

**Conclusion:**

Alcohol consumption is strongly correlated with having multiple sexual partners, sex with non-regular partner and engagement in transactional sex but not with consistent condom use at fish landing sites. However, the pattern and strength of this correlation differs by occupation. HIV risk reduction programs targeting the fishing communities should address alcohol consumption, particularly alcohol consumption before sexual contact. Different occupations may need different interventions.

## Background

The fishing communities are considered among the most at risk populations (MARPS) because of their high vulnerability to HIV infection and other sexually transmitted infections [1,2]. In many developing countries, fishing communities have HIV prevalence rates that are five to ten times higher than that of the general population and they lead highly risky sexual life [3]. A study among fishers in Thailand found that the HIV prevalence was 15%and 60% of them had multiple sexual partners [4]. A study among seafarers in Cambodia found that 60% had engaged in commercial sex and condom use was low [5]. The HIV prevalence among Cambodia fishermen was more than twice as much as it was in the general population [6]. In Uganda, a study carried out on Lake Albert, in the west of the country, found that nearly a quarter (24%) of members of the fishing community were HIV positive compared to 4% in neighbouring farming communities [3]. High HIV rates in the fishing communities are severely affecting fishers and related occupations already hit by falling fish stocks [1].

The high HIV prevalence in the fishing communities is believed to result from high levels of risky sexual behaviours which are in turn thought to be fuelled by high levels of alcohol abuse [7]. However, currently there is limited evidence to connect alcohol use and risky sexual behaviour in the fishing communities. Many studies in general populations have linked heavy alcohol consumption to lowered inhibition levels, fostering sexual risky behaviour such as multiple sexual contacts and a reduced likelihood of using condoms [8,9]. Without evidence one is bound to think that the relationship between alcohol use and risky sexual behaviour in the general population is the same as that in the fishing communities, yet the context of alcohol use in the fishing communities is distinctly different. For example, few in the general population are exposed to as much risk of death or harm and long working hours as the fishermen. This exposure to risk creates coping mechanisms that include heavy drinking and risky sexual behaviour [10]. Similar vulnerability to risky sexual behaviour has been observed among traders and service providers at fish landing sites. Poverty and declining fish stocks have led female traders into fish-for-sex competition, while service providers such as bar workers have resorted to commercial sex work to supplement their income [11]. A recent study found that the HIV prevalence was 29% among fishermen, 18-36% among male and female traders, and 43-46% among male and female service providers [12].

Heavy alcohol consumption and risky sexual behaviour are often presented as joint outcomes of risk taking behaviour among people in the fishing communities without critical analysis of the association between them. Alcohol consumption and transactional sex are shown as common habits in the fishing communities [8]. Having ample idle time [13], younger age (age 15–35 years) [7] and absence of family obligations among young single men [14] are associated with heavy alcohol consumption and risky sexual behaviour in this population. While alcohol consumption has been identified as one of the key drivers of infection and risky behaviour in the fishing communities [1,5,12,15], how risky sexual behaviour relates to different alcohol consumption patterns in this population remains largely unexplored in developing countries [11]. More scarce is information on how alcohol consumption patterns relate with risky behaviour in different occupational groups at fish landing sites.

Studies in the general population have shown that the frequency and quantity of alcohol consumption are highly correlated with the number of sexual partners and non-use of condoms [16]. A recent study in the state of Goa, India found that after controlling for demographics, the volume of consumption and frequency of heavy drinking predicted sexual risky behaviour [17]. A study in Finland found that frequent intoxication-related drinking increased the probability that teenagers had unprotected sex. Further, the likelihood of engaging in unprotected sex and/or having multiple sexual partners increased significantly with reports of intoxication [18]. Similar results were found in a systematic review of literature on studies in sub-Saharan Africa [19].

The number of drinking hours and day of drinking have been found to be directly or indirectly related to risky sexual behaviour in the general population. The number of drinking hours is closely related to intoxication and hence it can have an indirect impact on risky sexual behaviour. A study in Geneva found that intoxication hospitalisations fell by 25-40% as a result of restricted hours of drinking [20]. The day of the week when people drink is important in terms of how much they drink and likelihood of risky sexual behaviour. A study in the USA found that intoxication levels during celebratory events depended on day of drinking and motivation for the celebration [21].

The overall aim of this paper is to generate new knowledge that can be used in the fight against the effects of alcohol consumption as a risk sexual behaviour in the fishing communities. The health of the fishing communities is of high economic importance in Uganda’s economy since the fishing industry contributes 12% of the country’s GDP [22]. The sector employs 700,000 people directly and 1.2 million are totally or partially dependent on it [23].

## Methods

### Study setting and sample

The study was cross-sectional and targeted people at Kasenyi and Kigungu landing sites on Lake Victoria, both of which are within 50 km from Kampala, the country’s capital city. The respondents were aged 18–65 years. Both qualitative and quantitative data were collected but in this paper only quantitative data are reported.

Stratified sampling technique was applied in the selection of a sample to ensure representation of each of the three occupation categories of the fishing community, namely, fishermen, traders and service providers. Traders include those involved in selling of fish or their products and other small scale business at fish landing sites. Service providers include casual labourers, bar/restaurant attendants, commercial sex workers, and shop attendants. The chairpersons of fishermen and local council chairpersons of the landing sites helped in constructing provisional list of study participants for sampling and locating them for the study as well. In each occupation category a systematic random selection of respondents was carried out. Some data collectors, together with the local council chairpersons went close to the lake to interview the fishermen others went to the fish stalls and shops to get traders while the rest went to bars, eating places and households to interview service providers. The data collectors made sure that there was privacy before they conducted interviews. The interviews took around 45 minutes each.

Overall, the targeted number of respondents was 456 but there was oversampling and the number went up to 475. The targeted number of respondents in each group was meant to be 152 but there was over sampling among fish traders/operators (191) and under sampling among the fishermen (146) and service providers (138).

The sample size was computed using a formula by Levy and Lemeshow [24]. Considering 95% confidence interval of the results, probability of outcome of interest (having risky sexual behaviour) of 50%, an absolute difference (d) of 0.062 which we wanted to detect as significant and the population size of 300 for each stratum, the minimum sample size required was 137.

### Measures

Semi-structured questionnaires with questions covering background characteristics, alcohol use and sexual behaviour were administered by interviewers. The questions selected included those used to compute the alcohol use disorder test (AUDIT). The test comprises 10 questions which cover the domains of alcohol consumption, drinking behaviour, and alcohol-related problems [25]. In this study the AUDIT score was computed to identify the level of alcohol consumption and presence of drinking problems. However, only 9 of the questions were used in this study. The tenth question on whether the respondent or others had ever been injured as a result of her/his drinking was left out of the questionnaire by mistake. Each question is scored from 0 to 4 and scores for all questions are summed; total scores are categorized as 1–7, 8–15 and 16+ and represent levels of alcohol use disorder.

The indicators for HIV risky behaviour were: multiple sexual partners, non-use of condoms, inconsistent use of condoms, and transactional sex while those for alcohol use were frequency of consumption, AUDIT score, having got drunk in previous 30 days, length of time of drinking, days of drinking, and time for start of drinking. The consistency of questions on alcohol consumption as measured by scale reliability coefficient (Cronbach’s alpha) [26] was 53% while that for risky sexual behaviour indicators was 56%. The difference in coding may have contributed to a seemingly low consistency.

The outcomes of interest for risky sexual behaviour including having had more than one sexual partner, having had transactional sex, having unprotected sex (non-use of condom/inconsistent condom use) with non-spousal partner and having had sex with non-regular partners in previous 12 months. Independent variables of interest included frequency of alcohol consumption, alcohol use disorder identification test (AUDIT) score category, having got drunk in previous 30 days, usual length of time of drinking in hours, days of drinking in a week, and time of the day for start of drinking. The frequency of alcohol consumption was categorized into once a month, 2–4 times a month, and 2 or more times a week. The length of time of average drinking encounter was categorized as 0–2, 3–4 and 5 or more hours. The days of drinking were categorised into no specific day, weekdays or weekend only. The time for start of drinking was categorised into specific time versus any time of the day. In this paper having got drunk in previous 30 days is assumed to be a reflection of experience of drunkenness in previous 12 months.

### Data analysis

The data were analysed using STATA V10 software. The analysis process started with a description of socio-economic and demographic characteristics of the respondents and it was followed by the levels of alcohol consumption and risky sexual behaviour stratified by sex and occupation. The indicators of alcohol consumption used were: drank alcohol, drank 2 or more times a week, and got drunk in previous 30 days, and the AUDIT score category. Risky sexual behaviour indicators were: had more than 1 sexual partner in previous 12 months, had transactional sex in past 12 months, had non-regular partner in previous 12 months and had unprotected sex at last sex event. In the analysis that followed, alcohol consumption indicators were independent variables while risky sexual behaviour indicators were dependent or outcome variables. Analysis of condom use was restricted to sex with non-spousal partners because of low prevalence of condom use among married people. In the 2004/5 sero-survey only 3% of women and 4% of men were reported to have used condoms with their spouses [27]. Chi-square tests of significance were used to compare distribution of background characteristics, levels of drinking and risky sexual behaviour by sex.

The relationship between alcohol consumption and risky sexual behaviour was further explored in bivariate and multivariate logistic regression analysis. The bivariate analysis examined the relationship between each of the risky sexual behaviour indicators and alcohol consumption variables with separate analyses conducted for each occupation. The results for the bivariate analysis are not presented in this study but they were helpful in building multivariate models. Using an inclusion criterion of p < 0.1 in the bivariate analysis, independent variables were selected for the multivariate models. Using backward elimination procedure variables that had Wald’s test p-values higher than 0.05 in the multivariate models were excluded one by one. At the end, if the goodness of fit was not sufficient (Pearson’s p-value <0.05) some of the variables eliminated were included again. Tables showing odds ratios and their 95% confidence intervals from both bivariate and multivariate analysis are presented. Multivariate analysis could not be stratified for both sex and occupation because it would produce very wide confidence intervals as a result of small number of observations to cater for all variables in the models.

## Ethical clearance

In accordance with the Helsinki Declaration, the study was carried out after receiving ethical approval. The study was approved by the Makerere University School of Public Health Institutional Review Board and the Uganda National Council for Science and Technology. Before enrolment into the study, the respondents were informed about the aims of the study, their discretion to participate or withdraw at any time and were assured that all information obtained from them would be kept confidential. The anticipated benefits or risks of the study to the participants or the community were clearly explained and all the participants were given a chance to say whether they had understood the objectives of the study and what was expected of them as respondents. A question was as to whether they consented to participation in the study was asked.

## Results

### Characteristics of the respondents

The sample comprised of 475 respondents, of whom 303 (64%) were men and 172 (36%) were women (Table 1). Most of the respondents were young, as 91% were under 40 years of age. Most respondents had attained at least primary level of education, were Christians, had lived at the landing sites for more than one year and were within an income bracket of US$ 34–345 per month. The service providers were younger, single and mobile compared to the fishermen and traders. Other background characteristics did not significantly change by occupation. The service providers included commercial sex workers, bar maids and waiters or waitresses in eating places. Kasenyi landing site residents constituted two thirds of the respondents and the rest were resident at Kigungu landing site.

**Table 1:** Characteristics of respondents at Kasenyi and Kigungu fish landing sites

**Characteristic**	**All n (% of 475)**	**Fishermen n (% of 146)**	**Traders n (% of 191)**	**Service providers n (% of 138)**	**p-value†**
**Sex**					
Male	305(64.2)	146(100.0)	89(46.6)	50(50.7)	
Female	170(35.8)	0(0.0)	102(53.4)	68(49.3)	<0.001
**Age group**					
18-24	136(28.6)	31(21.2)	53(27.8)	52(37.7)	
25-29	152(32.0)	40(27.4)	66(34.6)	46(33.3)	0.003
30-39	144(30.3)	61(41.8)	51(26.7)	32(23.2)	
40+	43(9.1)	14(9.6)	21(11.0)	8(5.8)	
**Education**					
None	41(8.6)	14(9.6)	16(8.4)	11(8.0)	0.26
Primary	233(49.1)	81(55.5)	85(44.5)	67(48.6)	
Secondary	201(42.3)	51(34.9)	90(47.1)	60(43.5)	
**Marital status**					
Single	175(36.8)	51(34.9)	60(31.4)	64(46.4)	0.031
Married	231(48.6)	77(52.7)	102(53.4)	52(37.7)	
Other	69(14.5)	18(12.3)	29(15.2)	22(15.9)	
**Religion**					
Catholic	204(43.0)	61(41.8)	88(46.1)	55(39.9)	0.004
Protestant	121(25.5)	51(34.9)	35(18.3)	35(25.4)	
Muslim	91(19.2)	25(17.1)	35(18.3)	31(22.5)	
Other	59(12.4)	9(6.2)	33(17.3)	17(12.3)	
**Tribe**					
Baganda	277(58.3)	77(52.7)	125(65.5)	75(54.4)	0.13
Basoga	32(6.7)	12(8.2)	11(5.8)	9(6.5)	
Banyankore	45(9.5)	13(8.9)	13(6.8)	19(13.8)	
Other	121(25.5)	44(30.1)	42(22.0)	35(25.4)	
**Length of stay**					
< 1 year	90(19.0)	20(13.7)	31(16.2)	39(28.3)	
1-5	188(39.6)	62(42.5)	78(40.8)	48(34.8)	
>5	197(41.5)	64(43.8)	82(42.9)	51(37.0)	0.023
**Monthly income**					
<100,000 (US$34)	132(29.3)	29(20.1)	63(34.2)	40(32.5)	
100,000-199,000 (US$ 34–68)	110(24.4)	33(22.9)	46(25.0)	31(25.2)	0.026
200,000-999,999 (US$69-345)	177(39.3)	68(47.2)	61(33.2)	48(39.0)	
1 million+(US$346)	32(7.1)	14(9.7)	14(7.6)	4(3.3)	
**Site**					
Kigungu	160(33.7)	57(39.0)	61(31.9)	42(30.4)	
Kasenyi	315(66.3)	89(61.0)	130(68.1)	96(70.0)	0.25

† Chi-square p-value.

### Level of alcohol consumption and risky sexual behaviour

Table 2 shows the levels of alcohol consumption and risky sexual behaviour by occupation and sex. A higher proportion of men (61%) drank alcohol compared to the women (44%). Among the drinkers, 56% of men and 51% of women drank two or more times a week. Sixty two per cent of the male and 52% of the female drinkers said they got drunk in the previous 30 days. Among those who reported any alcohol use, a high proportion of men (69%) and women (68%) drank ‘any day’ and 32% of men and 33% of women drank ‘any time of the day’.

**Table 2:** Level of drinking and risky sexual behaviour at Kisenyi and Kigungu fish landing sites

**Drinking and sexual behaviour indicators**	**All n (%)**	**Fishermen n (%)**	**Traders n (%)**	**Service providers n (%)**	**p-value†**
**MEN**					
**Drinking††**					
Drinks alcohol	186(61.0)	96(65.8)	47(52.8)	43(61.4)	0.14
Drinks 2+ times a week	104(55.9)	62(64.6)	19(40.4)	23(53.5)	0.03
Got drunk in past 30 days	116(62.4)	74(77.1)	20(42.6)	22(51.2)	<0.001
AUDIT score>7	112(60.2)	67(69.8)	23(48.9)	22(51.2)	0.02
Drinks any day	128(68.8)	68(70.8)	33(70.2)	27(62.8)	0.81
Drinks anytime	60(32.3)	33(34.4)	13(27.7)	14(32.6)	0.70
Drinks for 3+ hours on a typical day	111(59.7)	70(72.9)	23(48.9)	18(41.9)	0.002
**Sexual behaviour (sexually active men)**					
Had >1 sexual partners in past 12mns	144(49.7)	77(53.5)	38(45.8)	29(46.0)	0.57
Had transactional sex in past 12mns	82(28.3)	48(33.3)	17(20.5)	17(27.0)	0.11
Had non-regular partner at last sex	60(20.7)	32(22.2)	16(19.3)	12(19.1)	0.81
Had unprotected sex in non-spousal relationship at last sex	91(62.8)	44(58.7)	23(67.7)	24(66.7)	0.50
All men	305(100)	146(100.0)	89(100)	70(100.0)	
**WOMEN**					
**Drinking††**					
Drinks alcohol	75(44.1)	--	38(37.3)	37(54.4)	0.03
Drinks 2+ times a week	38(50.7)	--	11(29.0)	27(73.0)	<0.001
Got drank in past 30 days	39(52.0)	--	14(36.8)	25(67.6)	<0.001
AUDIT score>7	41(54.7)	--	15(39.5)	26(70.3)	0.007
Drinks any day	51(68.0)	--	23(60.5)	28(75.7)	0.18
Drinks anytime	25(33.3)	--	7(18.4)	18(48.7)	0.004
Drinks for 3+ hours on a typical day	35(46.7)	--	19(50.0)	16(43.2)	0.84
**Sexual behaviour**		--			
Had >1 sexual partners in past 12mns	54(33.3)	--	21(21.2)	33(52.4)	<0.001
Had transactional sex in past 12mns	41(25.3)	--	17(17.2)	24(38.1)	0.003
Had non-regular partner at last sex	34(21.0)	--	13(13.0)	21(22.2)	0.002
Had unprotected sex in non-spousal relationship at last sex **†††**	39(59.1)	--	22(73.3)	17(47.2)	0.03
All women	170(100)	--	31(100)	36(100)	

††All questions that follow exposure to alcohol consumption apply to drinkers only. †Chi-sq p-value. mns means months ††† for only those that had non-spousal relationship at last sex.

Levels of alcohol consumption and risky sexual behaviour varied significantly by occupation. Compared to male traders and service providers, fishermen were more likely to drink frequently, get drunk, have higher AUDIT score, and drank for a much longer time but their levels of risky sexual behaviour did not change significantly. Among women, service providers were more likely to drink, drink more frequently, get drunk, have higher AUDIT score, drink any time of the day compared to traders. Female service providers were also more likely to have more than one sexual partner, engage in transactional sex, and have sex with a non-regular partner compared to traders. However, female traders were more likely to have unprotected sex (73%) compared to service providers (47%) (p=0.03).

### Relationship between alcohol consumption and HIV risky sexual behaviour by occupation in multivariate models

The relationship between each selected alcohol consumption indicator variable and each risky sexual behaviour was analysed using multivariate logistic regression by controlling for the demographic and background characteristics. This was carried out for each occupation.

Table 3 shows that among fishermen, only higher audit score was strongly associated with having had more than one sexual partner (OR=11.9, 95% CI: 3.00-47.0). However, all key independent factors –frequent drinking, higher audit score, getting drunk, long time of drinking, drinking any time and any day were strong correlates of engaging in transactional sex. Fishermen who drank 2 or more times a week were 7.9 times (95% CI: 2.05-30.24) more likely to have engaged in transactional sex previous 12 months than those who never drank alcohol. Those whose AUDIT score was 16 or more were 9 times (95% CI: 2.52-32.50) more likely to have engaged in transactional sex than those with lower scores. Those who got drunk in previous 30 days were 4.7 times (95% CI: 1.74-6.24) more likely to have engaged in transactional sex. None of the key independent factors was correlated with having had unprotected sex in non-spousal relationships. Higher AUDIT score was strongly correlated with having had sex with non-regular partner while having got drunk in previous 30 days and drinking any time of the day were only marginally correlated with the dependent variable.

**Table 3:** Relationship between each selected alcohol consumption indicator and each risky sexual behaviour among fishermen in a multivariate logistic regression

**Alcohol consumption patterns**	**Risky sexual behaviour**			
	**Had more than 1 sexual partners in past 12 months OR(95%CI)**	**Had transactional sex in past 12 months- OR(95%CI)**	**Never used/inconsistently used condom in non-spousal relationship OR(95%CI)**	**Had sex with non-regular partner at last sex OR(95%CI)**
**Taken alcohol in past 12 months (base=Never)**				
Once a month	3.44(0.72-16.31)	4.76 (0.66-34.11)	0.85(0.07-10.86)	4.42(0.91-21.57)
2-4 times a month	1.22(0.29-5.14)	1.64(0.30-9.16)	4.07(0.50-33.07)	3.69(0.94-14.45)
2+ times a week	3.06(1.00-9.36)*	7.88 (2.05-30.24)**	0.33(0.07-1.58)	2.21(0.31-15.77)
**AUDIT Score**^**a**^**(base=1-7)**				
8-15	1.04 (0.36-3.00)	5.14(1.54-17.14)**	0.05(0.00-0.67)	1.30(0.36-4.66)
16+	11.88 (3.00-47.04)**	9.04(2.52-32.50)**	1.60(0.21-12.21)	8.24(2.39-28.37)**
**Got drunk in past 30 days - (Base=No)**				
Yes	2.10 (0.88-5.01)	4.74(1.62-13.93**	1.05 (0.22-5.02)	2.95(1.01-8.66)*
**Length of time of drinking (Base 0–2)**				
3-4 hours	0.37(0.08-1.71)	2.40 (0.50-11.62)	0.06 (0.00-1.58)	4.74(0.35-63.84)
5+ hours	0.78 (0.15-4.22)	11.78(2.01-68.89)*	0.06 (0.00-1.62)	---
**Days of drinking (base=Weekends only)**				
Any day	2.27 (0.64-8.11)	5.56 (1.41-21.95)*	0.92(0.18-4.76)	4.30(0.84-22.12)
**Time for start of drinking (specific time)**				
Anytime	1.99(0.63-6.25)	3.81 (1.30-11.18)*	0.27(0.05-1.59)	3.96(1.05-14.94)*

^a^The AUDIT score is based on 9 of 10 questions. *=p<0.05 **= p<0.01 ***= p<0.001 than 5 in some cells.

Table 4 shows the relationship between each selected alcohol consumption indicator and each risky sexual behaviour among traders in multivariate model. The results show that, frequent alcohol consumption, higher AUDIT score, getting drunk and drinking on any day of the week were strong correlates of having had more than one sexual partner in previous 12 months. Those who got drunk in previous 30 days were 18 times more likely to have had more than 1 sexual partner in previous 12 months (95% CI: 6.24-53.24). Significant correlates of engaging in transaction sex were frequent alcohol consumption, higher AUDIT score, getting drunk, long time of drinking and drinking at any time of the day. Traders who drank 2 or more times a week were 10 (OR=2.20-31.68) times more likely to have had transactional sex in previous 12 months. Like fishermen, none of the key independent factors was correlated with unprotected sex among traders. Factors correlated with having sex with a non-regular partner were higher AUDIT score and getting drunk. A traders with higher AUDIT score (16+) was 13 (95% OR: 2.70-66.47) times more likely to have had sex with a non-regular partner at last sex encounter.

**Table 4:** Relationship between each selected alcohol consumption indicator and each risky sexual behaviour among traders in a multivariate logistic regression

**Alcohol consumption patterns**	**Risky sexual behaviour**			
	**Had more than 1 sexual partner in past 12 months OR(95%CI)**	**Had transactional sex in past 12 months- OR(95%CI)**	**Never used/inconsistently used condom in non-spousal relationship OR(95%CI)**	**Had sex with non-regular partner at last sex OR(95%CI)**
**Taken alcohol in past 12 months (base=Never)**				
Once a month	5.53(1.83-16.67)**	2.91 (0.81-10.43)	0.36(0.00-50.39)	0.05(0.00-1.39)
2-4 times a month	9.01(2.22-36.49)**	1.70(0.38-7.64)	0.10(0.00-2.18)	0.38(0.03-4.49)
2+ times a week	4.79(1.48-15.50)**	8.35(2.20-31.68)**	0.76(0.03-22.82)	3.12(0.65-14.91)
**AUDIT Score**^**a**^**(base=1-7)**				
8-15	12.71(3.63-44.43)***	7.77(2.98-20.30)***	0.66(0.04-9.79)	2.12(0.62-7.18)
16+	8.00(1.54-44.51)*	10.31(2.69-39.50)**	0.43(0.02-11.79)	13.39(2.70-66.47)**
**Got drunk in past 30 days - (Base=No)**				
Yes	18.22(6.24-53.24)***	11.45(4.56-28.73)***	1.33(0.08-21.42)	5.12(1.23-21.16)*
**Length of time of drinking (Base 0–2)**				
3-4 hours	4.88(0.97-24.65)	2.58(0.67-9.86)	1.87(0.27-13.03)	1.27(0.24-6.46)
5+ hours	3.40(0.46-25.01)	8.07(1.97-33.00)**	2.85(0.20-41.06)	3.47(0.66-18.19)
**Days of drinking (base=Weekends only)**				
Any day	3.13(1.12-8.75)*	1.19(0.41-3.45)	0.29(0.05-1.79)	0.76(0.20-2.91)
**Time for start of drinking (specific time)**				
Anytime	2.28(0.45-10.93)	6.93(1.40-34.30)*	0.52(0.08-3.54)	1.93(0.47-7.88)

^t^The AUDIT score is based on 9 of 10 questions. *=p<0.05 **= p<0.01 ***= p<0.001.

Table 5 shows the association between individual alcohol consumption indicators and risky sexual behaviour among service providers in a multivariate model. Frequent alcohol consumption, higher AUDIT score (16+), getting drunk and long drinking time (5+ hours) were strong correlates of having had more than 1 sexual partner. Service providers who took alcohol 2 or more times a week were 6 times (95% CI: 2.44-16.54) more likely to have had more than 1 sexual partner compared to those who never took alcohol. Factors associated with having engaged in transactional sex in past 12 months were frequent alcohol consumption, higher AUDIT score, getting drunk, long drinking hours and drinking at any time of the day. Service providers who drank any time of the day were 6.5 times (95% CI: 1.94-22.01) likely to engage in transactional sex compared to those who had definite time of drinking. None of the independent factors investigated was correlated with having unprotected sex and having sex with a non-regular partner.

**Table 5:** Association between individual alcohol consumption indicators and risky sexual behaviour among service providers in multivariate logistic regression

**Alcohol consumption patterns**	**Risky sexual behaviour**			
	**Had more than 1 sexual partners in past 12 months - OR(95%CI)**	**Had transactional sex in past 12 months OR(95%CI)**	**Never used/inconsistently used condom in non-spousal relationship (95%CI)**	**Had sex with non-regular partner at last sex- OR(95%CI)**
**Taken alcohol in past 12 months (base=Never)**				
Once a month	0.46(0.09-2.45)	0.54(0.06-4.92)	0.58(0.06-5.27)	0.25(0.03-1.97)
2-4 times a month	9.80(2.21-43.59)**	3.61(0.89-14.62)	0.84(0.15-4.62)	0.43(0.05-3.85)
2+ times a week	5.68(2.21-4.62)***	6.35(2.44-16.54)***	1.18(0.35-3.93)	1.05(0.27-4.03)
**AUDIT score**^**a**^**(base=1-7)**				
8-15	1.96(0.75-5.09)	0.91(0.31-2.70)	1.49(0.37-6.02)	0.60(0.18-2.03)
16+	8.48(2.30-31.26)**	8.65(2.62-28.57)***	1.90(0.45-7.99)	2.85(0.90-9.02)
**Got drunk in past 30 days - (Base=No)**				
Yes	4.34(1.88-10.10)**	4.39(1.92-10.01)***	0.48(0.15-1.51)	2.30(0.94-5.60)
**Length of time of drinking in hours - (Base 0–2)**				
3-4	4.00(0.74-21.61)	2.42(0.52-11.17)	0.72(0.07-7.44)	2.68(0.41-17.50)
5+	6.04(1.40-25.98)*	5.78(1.50-22.24)*	1.16(0.14-9.39)	4.30(0.88-21.08)
**Days of drinking (base=Weekends only)**				
Any day	2.50(0.82-7.63)	6.39(1.60-25-52)	0.23(0.02-2.12)	1.36(0.33-5.70)
**Time for start of drinking (specific time)**				
Anytime	1.16(0.39-3.49)	6.54(1.94-22.01)**	0.21(0.03-1.49)	2.99(0.83-10.81)

^a^The AUDIT score is based on 9 of 10 questions. *=p<0.05 **= p<0.01 ***= p<0.001.

## Discussion

This paper fills a knowledge gap on relationship between alcohol consumption and risky sexual behaviour, namely number and type of sex partners, use of condoms, and engagement in transactional sex, among subpopulation living and working in fish landing environments. It has addressed key research questions on the level of alcohol consumption and risky sexual behaviour, nature and strength of relationship between alcohol consumption and risky sexual behaviour in this setting, and provided clues on possible areas of intervention.

The levels of harmful use of alcohol and risky sexual behaviour at the fish landing sites are higher than what has been found in previous studies. For example, according to the 2001 demographic and health survey, a half of male and a quarter of female drinkers got drunk within the previous 30 days [28]. This is much lower than 62% of male and 52% of female drinkers that got drunk in the same period in this study.

The high level of risky sexual behaviour fits the categorization of most at risk population (MARPS). The prevalence of risky sexual behaviour at the landing sites is much higher than that found in the general population. According to the 2004/5 national HIV sero-prevalence behavioural survey the proportion of sexually active respondents that had had 2 or more partners in previous 12 months was 29% among men and 4% among women [27], but in this study it was 50% among men and 33% among women. In the same survey, 0.5% of women and 1% of men said they engaged in transactional sex, but in this study shows that 28% of men and 25% of women were engaged in transactional sex.

Higher odds of engaging in risky sexual behaviour with higher frequency of alcohol consumption is consistent with many general population studies in different countries including those carried out in the USA, Finland and sub-Saharan Africa [16-19]. The correlations between alcohol consumption and risky sexual behaviour show that reduced alcohol consumption may reduce risky sexual behaviour. The correlations between the AUDIT score and risky sexual behaviour suggest that reduced odds of risky sexual behaviour may not only be realized with reduced frequency of alcohol consumption but also with control of all alcohol use disorders.

The experience of intoxication is a proxy measure for amount drunk. Its strong relationship with risky sexual behaviour in this study, reflects what has already been established in a population based study in Uganda [29]. This finding relates closely with the previous results on correlations between risky sexual behaviour and both AUDIT score and frequency of alcohol consumption.

A literature search shows that few studies have investigated the correlation between risky behaviour and length of time, day of the week and time of the day of drinking in the region and Africa as a whole. This is one area in which this study makes a major contribution to this field of knowledge. Evidence of reduced odds of risky sexual behaviour among those who drink for a short time, drink on a specific day of the week and specific time of the day calls for disciplined alcohol consumption and increased control of access to alcohol as strategies for promoting safer sexual behaviour.

Results on non-significant correlation between inconsistent/non-use of condom with alcohol consumption patterns are in agreement with several other studies. A meta analysis carried out in 2002 found that drinking is not necessarily linked to unprotected intercourse; the relationship between alcohol use and unprotected sex depends on sexual experience of the partners [30]. Given that the respondents were adults with long time exposure to both sexual activity and alcohol consumption the relationship may not be significant as results of the 2002 meta analysis showed. Other studies carried out in 2000 and 2002 showed less support for increased risk behaviour as a result of alcohol consumption [31,32]. However, there are still many studies that support the relationship between alcohol use and inconsistent or non-use of condom use [33].

The results have further shown that, for each occupation there is a unique set of modifiable alcohol consumption indicators that are correlated to particular risky sexual behaviour indicators. For example, among the fishermen frequent alcohol consumption, higher AUDIT score, getting drunk, longer time of drinking, days of drinking and time for drinking are all strongly correlated to engagement in transactional sex but not with other sexual behaviour indicators. Among traders, it is five of the six alcohol consumption indicators that are correlated with engagement in transactional sex while three of the factors are correlated with having more than one sexual partner. Among service providers four of the alcohol factors are correlated with having more than one sexual partner while five of the factors are correlated with having had transactional sex.

### Limitations

This study is subject to several limitations. First, the findings from two landing sites on Lake Victoria in Uganda may have limited generalizability to landing sites in other settings, such as those less close to an urban centre such as Kampala city. Second, due to the cross-sectional nature of this study, causal relationships for alcohol consumption and risky sexual behaviour cannot be established. Third, alcohol consumption and sexual risk behaviour may have been underreported since they were measured by self-reporting in a single survey and may also be subject to socially desirable responses; alternative methods, such as the Bogus Pipeline Method could potentially have increased the validity of self-report of alcohol consumption [34]. In this method, the person whose attitude or emotion is being measured is told that they are being monitored by a machine or a polygraph (lie detector), resulting in more truthful answers [35].

Having got drunk in previous 30 days was taken to be a proxy measure of having got drunk in previous 12 months but this may not be a right measure for people who drink occasionally. Some people may not have got drunk in previous 30 days because there was no event that could expose them to alcohol consumption but they drank in a month preceding the previous one.

## Conclusions

The study findings indicate that the levels of alcohol consumption and HIV risky sexual behaviours in communities at fish landing sites of Lake Victoria are quite high. There is a strong correlation between indicators of alcohol consumption and those of HIV risky sexual behaviour. In some instances the higher the extent of alcohol use the more the likelihood of risky sexual behaviour. The screening for alcohol consumption and risky sexual behaviours was feasible. Research is needed to enhance cultural understanding of alcohol consumption and risky sexual behaviours in the fishing communities. Combined interventions to educate and control access to alcohol in fishing communities may be able to reduce alcohol consumption and sexual risk behaviours.

Correlation of different sets of alcohol consumption indicators with particular risky behaviour variables in each kind of occupation shows a need for formulating different interventions in each occupation.

An intervention study involving control of sale of alcohol coupled with health education that promotes disciplined alcohol consumption is recommended as a follow-up to this work.

## Competing interests

The authors declare that they have no competing interest.

## Authors’ contributions

NMT conceived the idea and designed the study, guided the data collection, analysed the data, and led the writing process. LA contributed substantially in design, data collection, analysis and writing. RKW contributed greatly at the writing stage, SPSK contributed to the design of the study, data analysis and writing. QL contributed to the writing. FWM contributed to the concept and design. GW contributed to the concept, design and writing. All authors read and approved the final manuscript.

## Pre-publication history

The pre-publication history for this paper can be accessed here:

http://www.biomedcentral.com/1471-2458/12/1069/prepub
